# Identification of MMV Malaria Box Inhibitors of *Perkinsus marinus* Using an ATP-Based Bioluminescence Assay

**DOI:** 10.1371/journal.pone.0111051

**Published:** 2014-10-22

**Authors:** Yesmalie Alemán Resto, José A. Fernández Robledo

**Affiliations:** 1 Bigelow Laboratory for Ocean Sciences, Boothbay, Maine, United States of America; 2 Research Experiences for Undergraduates (REU) NSF Program - 2013 - Bigelow Laboratory for Ocean Sciences, Boothbay, Maine, United States of America; Bernhard Nocht Institute for Tropical Medicine, Germany

## Abstract

“Dermo” disease caused by the protozoan parasite *Perkinsus marinus* (Perkinsozoa) is one of the main obstacles to the restoration of oyster populations in the USA. *Perkinsus* spp. are also a concern worldwide because there are limited approaches to intervention against the disease. Based on the phylogenetic affinity between the Perkinsozoa and Apicomplexa, we exposed *Perkinsus* trophozoites to the Medicines for Malaria Venture Malaria Box, an open access compound library comprised of 200 drug-like and 200 probe-like compounds that are highly active against the erythrocyte stage of *Plasmodium falciparum*. Using a final concentration of 20 µM, we found that 4 days after exposure 46% of the compounds were active against *P. marinus* trophozoites. Six compounds with IC_50_ in the µM range were used to compare the degree of susceptibility *in vitro* of eight *P. marinus* strains from the USA and five *Perkinsus* species from around the world. The three compounds, MMV666021, MMV665807 and MMV666102, displayed a uniform effect across *Perkinsus* strains and species. Both *Perkinsus marinus* isolates and *Perkinsus* spp. presented different patterns of response to the panel of compounds tested, supporting the concept of strain/species variability. Here, we expanded the range of compounds available for inhibiting *Perkinsus* proliferation *in vitro* and characterized *Perkinsus* phenotypes based on their resistance to six compounds. We also discuss the implications of these findings in the context of oyster management. The *Perkinsus* system offers the potential for investigating the mechanism of action of the compounds of interest.

## Introduction

*Perkinsus marinus* and *Perkinsus chesapeaki* cause Dermo disease in oysters and clams in the USA. Described in the early 1950s, Dermo disease is associated with mass mortalities of eastern oysters (*Crassostrea virginica*) in the Gulf Coast [1]; now it is under surveillance by the World Organization for Animal Health (OIE; http://www.oie.int/; Aquatic Animal Health Code, Section 11: Diseases of Molluscs). The Chesapeake Bay (Maryland, Virginia, USA) is a clear example of where *P. marinus* has contributed to the decimation of the oyster industry (today's production in Maryland is 4.2% of the production in the mid-1960s). The expansion of the *P. marinus* distribution range in the USA has been associated with global warming and the shellfish trade [2], [3]. Dermo remains an important obstacle to the restoration of oyster populations in numerous eastern states [3], [4]. Interestingly, *P. marinus* has also been reported with high prevalence in oysters from eastern states with no noticeable mollusk mortality [5], and recent records of *P. marinus* in oysters from the West Coast of North America were not associated with mortalities [6]. The presence of *P. marinus* phenotypes and genotypes might account for differences in virulence [7]–[9]. In the USA, *P. chesapeaki* displays a high preference for infecting clams and it appears to be better adapted to lower salinities and temperatures than *P. marinus* [5] and recently it has been detected in cockles (*Cerastoderma edule*) in Europe [10]. Worldwide, seven *Perkinsus* spp. have been described, most of them in the last decade with five of them available in *in vitro* culture (reviewed in [11]).

Compared to parasites of human and veterinary relevance, the pharmacopoeia for marine protozoan parasites is still very limited, and some of these compounds are toxic in the marine environment [12], [13]. *Perkinsus* and other non-photosynthetic relatives of both dinoflagellates and apicomplexans lineages have lost the ability to perform photosynthesis; still, they have retained a cryptic plastid and its pathways (Chromalveolata hypothesis), which are recognized as promising drug targets [14], [15]. Government agencies, drug companies, and non-profit organizations have screened multiple compound libraries against *Plasmodium falciparum* resulting in the Medicines for Malaria Venture (MMV) Malaria Box (http://www.mmv.org/malariabox) [16]. This compound library is being used to find inhibitors of defined parasite life stages [17], [18], to describe mechanisms of action [19], and to find active compounds against other protozoan parasites [20], [21]. Here, we followed similar approach and tested the MMV Malaria Box for the discovery of novel hits against *Perkinsus* using an adenosine tri-phosphate (ATP) content-based assay to test *P. marinus* proliferation growth [13].

## Materials and Methods

### Materials

The MMV Malaria Box constitutes 200 drug-like and 200 probe-like compounds with activity against the blood-stage of *Plasmodium falciparum* 3D7 (http://www.mmv.org/research-development/malaria-box-supporting-information). Stock solutions (20 mM) (Batch April2013; Table S1) in dimethyl sulfoxide (DMSO) were diluted in water and tested in the primary screening at a final concentration of 20 µM. The compounds were not repurchased nor re-synthetized; consequently, the results should be considered as primary unconfirmed hits until the identification of these compounds is followed up by a proper confirmation.

### Parasite strains and *in vitro* culture

Experiments were carried out with eight *P. marinus* strains and five *Perkinsus* species (Table 1). Cultures were maintained in Dulbecco modified Eagle's: Ham's F12 (1∶2) supplemented with 5% fetal bovine serum in 25 cm^2^ (5 ml) vented flasks in a 26–28°C microbiology incubator as reported elsewhere [22]. For the compound library screening, *P. marinus* PRA240 [13], [23] cultures were expanded in a 75 cm^2^ (30–50 ml) vented flask in a microbiology incubator fitted with orbital shaking (70–80 rpm).

**Table 1:** *Perkinsus* spp. and *Perkinsus marinus* strains used in the study.

*Perkinsus* sp.*	Strain	ATCC #	Location/year of isolation	Host	Reference
*P. marinus*	C13-11 [MA-2-11]	50896	Cotuit, MA (USA)/1998	*Crassostrea virginica*	
	LICT-1 [CT-1]	50508	Long Island Sound, CT (USA)/1998	*Crassostrea virginica*	
	DBNJ-1 [NJ-1]	50509	Delaware Bay, NJ (USA)/1993	*Crassostrea virginica*	
	CB5D4	PRA240	Bennett Point, MD (USA)/2008	*Crassostrea virginica*	[23]
	CB5D4	PRA393	GFP mutant derived from PRA240	*Crassostrea virginica*	[13], [23]
	HCedar2	50757	Cedar Keys, FL (USA)/1998	*Crassostrea virginica*	
	HTtP14 [FL-6]	50763	Fort Pierce, FL (USA)	*Crassostrea virginica*	
	TXsc	50983	Galveston Bay, TX (USA)/1993	*Crassostrea virginica*	[28]
*P. chesapeaki* ( = *andrewsi*)	A8-4a	50807	Fox Point, MD (USA)/2001	*Macoma balthica*	[25]
*P. olseni* ( = *atlanticus*)	ALG1	50984	Ria Formosa, Algarve (Portugal)/2002	*Tapes decussatus*	[24]
*P. mediterraneus*	G2	PRA238	Menorca (Spain)/2003	*Ostrea edulis*	[47]
*P. honshuensis*	Mie-3G/H8	PRA177	Gokasho Bay, Mie Pref. (Japan)/2002	*Venerupis philippinarum*	[56]

*Perkinsus marinus* PRA240 was used for the primary screen. A total of eight *P. marinus* strains isolated from oysters from the East and Gulf Coast of the USA and five *Perkinsus* spp. from around the world were used for the secondary screen. In all the cases cultures were maintained in Dulbecco modified Eagle's: Ham's F12 (1∶2) supplemented with 5% fetal bovine serum.

*Perkinsus qugwadi and *Perkinsus beihaiensis* have never been available in culture [57], [58].

### 96-Well format *Perkinsus* growth-inhibition primary screen

*Perkinsus marinus* PRA240 (100 µl, 2.0–5.0×10^6^ cells/ml or 2000–4000 relative fluorescence units, RFU) were prepared in sterile 96-well plates (white OptiPlateTM-96, PerkinElmer Life Sciences, Boston, MA). *Perkinsus marinus* cells were exposed once to the MMV Malaria Box (20 µM; final concentration of DMSO was 0.1%; concentrations above 0.1% are toxic to *P. marinus* trophozoites) in triplicate. Control wells (×3) included DMSO with *Perkinsus* cells, culture medium with cells and culture medium with no cells. The effect of the compounds on *P. marinus* proliferation was evaluated using the ATPlite assay at day 4 post-exposure, as reported elsewhere [13]. Readings for each well were normalized to the control wells with cells and DMSO (100% activity).

### Secondary *Perkinsus* growth-inhibition screen (IC_50_) and *Perkinsus* strain and species sensitivity

Six of the best hits from the MMV Malaria Box (Table 2) were retested on *P. marinus* PRA240; the IC_50_ was calculated in an 8-point dose-response curve (10 µM to 0.156 µM) using Prim6 (sigmoidal) (Graphpad Software, Inc.). Eight *P. marinus* strains and five *Perkinsus* spp. (Table 1) were tested to compare their relative sensitivity using 2 µM day 2 post-exposure. *In vitro* cultures *Perkinsus olseni*, *P. chesapeaki*, and *P. mediterraneus* are characterized by either sporulating, making the culture medium acid or remaining in clumps, or having very large trophozoites [24]–[27]. Consequently, to standardize the assay, aliquots from the cultures in the exponential phase were used for ATP measurement and then the experimental-well plates seeded with cells- ATP activity equivalent to *P. marinus* PRA240 2.0–5.0×10^6^ cells (2000–4000 RLU) [13]. The effect of the compounds on *P. marinus* strains and *Perkinsus* spp. proliferation was evaluated as above.

**Table 2:** List of compounds active against *Plasmodium falciparum* selected for the MMV Malaria Box (http://www.mmv.org/malariabox) for secondary *Perkinsus marinus* growth-inhibition screen (IC_50_) and *Perkinsus marinus* strain and *Perkinsus* species sensitivity.

HEOS Compound ID	Target	Smiles	EC_50_ (µM)*	Set	MW (KDa)	EC_50_ (µM)**
MMV665941	Unknown	CN(C)c1ccc(cc1)C(O)(c2ccc(cc2)N(C)C)c3ccc(cc3)N(C)C	0.255	Probe-like	389.53	5.35
MMV666021	Yes, 29	Cc1ccc(cc1)c2cc3C( = O)c4ccccc4c3nn2	0.094	Probe-like	272.30	1.05
MMV665807	TM protease serine 4	Oc1ccc(Cl)cc1C( = O)Nc2cccc(c2)C(F)(F)F	ND	Drug-like	315.67	2.00
MMV666102	Functional 17	CN(C)c1ccc(cc1)c2nc3cc(N)ccc3[nH]2	ND	Drug-like	252.31	1.77
MMV396719	Functional 11	n1(c2c(cccc2)n3)c3c4c(cccc4)NC1(C)c5cccc(OC)c5	1.150	Drug-like	341.40	2.08
MMV006522	Functional 19, Cytotoxic	CCOc1ccc2nc(C)cc(Nc3ccc(Br)cc3)c2c1	0.480	Probe-like	357.24	35.61

*Plasmodium falciparum 3D7;

**Perkinsus marinus PRA240 primary screen.

## Results and Discussion

### MMV Malaria Box screen

**Figure 1:**
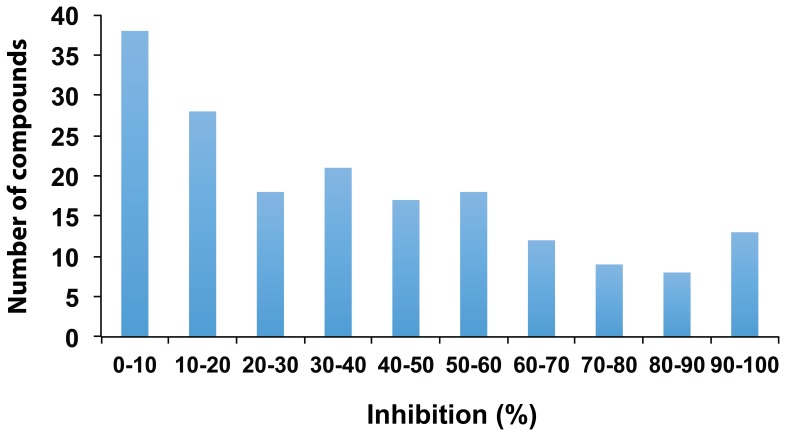
Percentage of inhibition of *Perkinsus marinus* using the MMV Malaria Box. Biological triplicate cultures were grown in sterile 96-well plates (100 µl; 2.0×10^6^ cells/ml) and cells were exposed to the MMV Malaria Box (20 µM). The effect of the drugs on *P. marinus* proliferation was evaluated using the ATPlite assay at day 4 post-exposure to the selected drugs. Readings for each concentration were normalized to the control wells with each solvent (100% activity). A total of 122 (67.0%) compounds resulted in at least 50% inhibition; from these compounds, 13 (7.1%) resulted in at least 90% inhibition (Figure 2).

**Figure 2:**
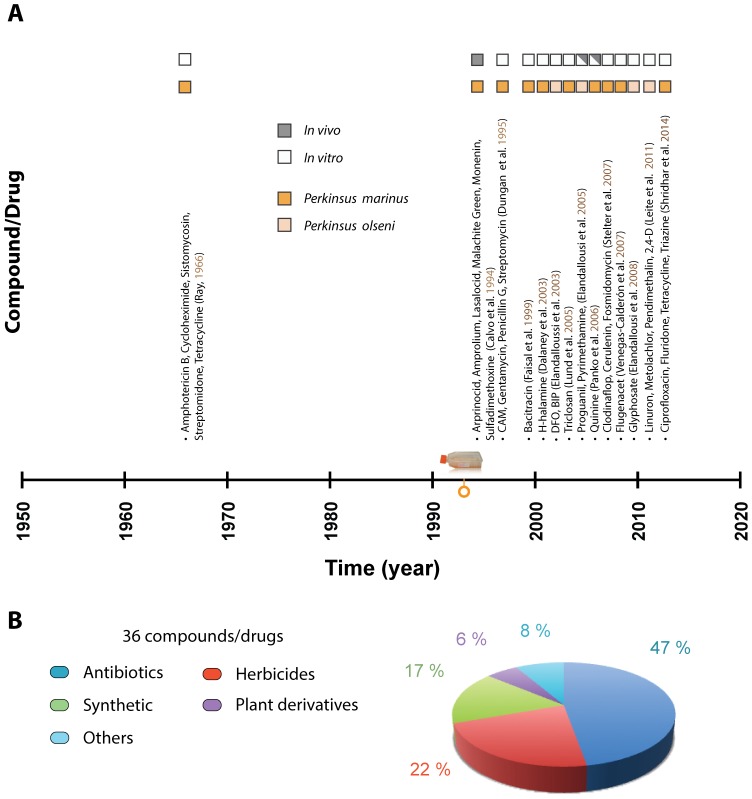
Drug discovery against Dermo disease. (A) Time line for the discovery of drugs against Dermo disease, starting when the etiological agent was described until this study. Most of the discoveries did happen after the development of the culture methodologies for *Perkinsus* spp. in 1993 and most studies have been carried out in *in vitro* cultures. (B) Percentage of the compounds active against *Perkinsus* based on their chemical nature. (C) Percentage of available compounds against Dermo tested in *Perkinsus marinus* and *Perkinsus olseni*.

In this study, we screened the MMV Malaria Box for compounds that might inhibit *P. marinus* proliferation *in vitro*, an approach that has been successfully used to identify compounds against other protozoan parasites [19]–[21]. In our previous study, the effect of the drugs on *P. marinus* proliferation was evaluated at days 2, 4, and 8 post-exposure; however, it was at day 4 post-exposure when the inhibitory effect(s) of most drugs tested became apparent [13]. Consequently, for the MMV Malaria Box screening we measured cell viability at day 4 post-exposure. We found that 46% of the compounds active against the *P. falciparum* erythrocyte life stage were also active against *P. marinus* trophozoites (Table S2). A total of 58 compounds (31.8%) resulted in at least 50% inhibition; from these compounds, 13 (7.1%) resulted in at least 90% inhibition (Figure 1). The repertory of available anti- *Perkinsus* drugs has gradually increased over the past two decades thanks to the establishment of the culture methodologies for *Perkinsus* spp. [28]–[30] (Figure 2A). Still, prior to this study, the number of available compounds against *Perkinsus* spp. was very limited (Figure 2B) compared to compounds against protozoan parasites of medical and veterinary relevance [31]–[34]. Previous screenings for compounds inhibiting *Perkinsus* proliferation have been based on the strong line of evidence for the presence in *Perkinsus*, like those in apicomplexan parasites, of pathways linked to a relic plastid [12], [13], [35], [36]. Here we have shown that the MMV Malaria Box offers a promising alternative way of finding compounds effective against *Perkinsus* spp.

### Secondary *Perkinsus* growth-inhibition screen (IC_50_)

**Figure 3:**
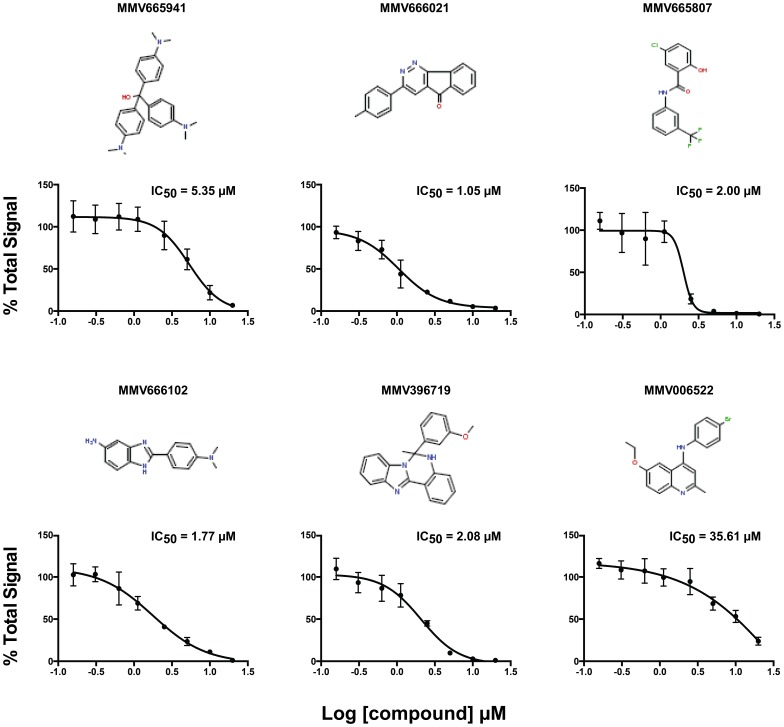
Secondary *Perkinsus marinus* growth-inhibition screen (IC_50_). Biological triplicate cultures were exposed to an 8 -point dose-response curve (10 µM to 0.156 µM). The effect of the drugs on *P. marinus* proliferation was evaluated as above.

Three drug-like and three probe- like of the 13 compounds with the highest inhibitory effect on *P. marinus* (Table 2) were randomly selected for calculating the IC_50_ in an 8-point dose-response curve (10 µM to 0.156 µM). We found that the IC_50_ varied between 1.05 µM for MV66602 and 5.35 µM for MMV665941; for MV006522 the IC_50_ was 35.6 µM a high concentration or leaving the compound longer time would have resulted in a fitted sigmoidal curve. In this study the IC_50_ for the selected compounds (Figure 3) was in the lower µM range and much lower than for the compounds tested in our previous study [13], still it was higher than the corresponding *P. falciparum* IC_50_ values (Table 2); consequently, without knowing the mechanism of action of the compounds, we cannot rule out off-target effects due to non-specific cytotoxic agents, including detergent effects, multi-targeting and oxidative effects. The nature of the assays (*Plasmodium falciparum* relies on infected erythrocytes and the *P. marinus* screen is performed in the absence of the host cells) and culture medium can also account for the differences in the IC_50_ values. With a direct life cycle, *P. marinus* trophozoites are phagocytized by the oyster hemocytes [37], [38] where they resist oxidative killing [39]. Interestingly, MMV666021 has been involved in the inhibition of glutathione-S-transferase (GST) activity of prostaglandin D2 synthase (PGDS) [40]. GST are involved in parasite survival by protecting them against oxidative stress from the host or from products derived from their own metabolism [41], and in *P. falciparum* it has been associated with chloroquine-resistance [42]. We grow *Perkinsus* in a host cell-free culture medium; hence, if the MMV666021 is indeed affecting the oxidative stress, it is most likely dealing with the ROS derived from the parasites' own metabolism. *Perkinsus marinus* trophozoites have an expanded transporter repertoire, which is useful not only for transporting nutrients but also for secreting extracellular products (ECP) intended to inactivate the host defense and to break down host tissues [43], [44]. Protease activity variations significantly decrease the migration of hemocytes [44] and have been associated with differences in the average cell size and growth rate [45]. MMV665807 is believed to target transmembrane serine proteases. Interestingly, the *P. marinus* genome encodes multiple putative serine protease genes (*e.g.* XM_002788359, XM_002786609, XM_002766692); numerous studies have identified serine protease activities in the spent medium of cultured *P. marinus* and *P. mediterraneus* [46], [47] and mutations in the promoter region of serine protease inhibitors (SPIs) in *C. virginica* which confer resistance to Dermo disease [48], [49]. The parasite proteases could be the target of MMV665807; however, to prove this hypothesis would require further experiments outside the scope of this study.

### *Perkinsus marinus* strains and Perkinsus species sensitivity

Our results comparing the effect of a single compound concentration (2 µM) against seven *P. marinus* strains indicate that for MMV666021, MMV665807 and MMV666102, the inhibition of the different strains was within an equivalent range (Table 3). Interestingly, for MMV665941, MMV396719 and MMV006522, there was a high degree of variability among the *P. marinus* strains. The presence of *Perkinsus* in low salinity estuaries and the sudden spread of the parasite between oyster beds is often seen as indicating the presence of strains adapted to low salinity and strains of variable virulence; parasites isolated from the Atlantic coast are more virulent than Gulf isolates [50], [51]. Indeed, *P. marinus* “races” and genetic strains have been documented along the Atlantic and Gulf coasts of the USA [7]–[9]. *Perkinsus marinus* strains from Maryland and Virginia appeared to be more susceptible to treatment with the antimalarial drug Quinine (130 µM) [52]. We also found strain variability to the compounds tested; *P. marinus* HCedar 2 from Florida appears to be much more sensitive to MMV006522 than the other *P. marinus* strains. On the other hand, MMV665941 appears to be less effective against *P. marinus* LICT-1 [CT-1] and DBNJ-1 [NJ-1], isolated from oysters from Connecticut and Delaware respectively. *Perkinsus marinus* LICT-1 [CT-1] also appears to be the strain less sensitive to MMV396719. There is a genetic base beneath *P. marinus* strains [8], [9] and recent microsatellite analyses suggest that *P. marinus* utilizes both sexual and asexual reproduction and, over the short-term, selection acts upon independent parasite lineages rather than upon individual *loci* in a cohesive, interbreeding population [53]. Drawing a parallel to other protozoan parasites with markedly clonal population structure and variable degree of virulence [54], it is possible that the observed variability respond to a clonal population structure (strains derived from one original single clone) with variable virulence. Indeed, Reece et al. [8] grouped 76 *P. marinus* isolates in 12 different composite genotypes with >88% of isolates possessing one of three predominant genotypes.

**Table 3:** Activity of antimalarial drug-like and probe-like (2 µM) compounds on *P. marinus* strains and *Perkinsus* spp. determined at day 2 post-exposure.

			HEOS Compound ID											
			MMV665941		MMV666021		MMV665807		MMV666102		MMV396719		MMV006522	
Species	Strain		Mean	SDV	Mean	SDV	Mean	SDV	Mean	SDV	Mean	SDV	Mean	SDV
*P. marinus*	C13-11 [MA-2-11]	Massachusetts	34.9	1.9	86.3	1.5	97.1	0.8	74.5	1.6	39.7	7.7	−10.3	0.2
	LICT-1 [CT-1]	Connecticut	−20.7	10.2	79.6	1.6	95.6	0.2	64.6	1.9	36.7	10.2	−4.6	7.7
	DBNJ-1 [NJ-1]	Delaware	−6.8	11.0	71.6	3.2	92.4	1.1	72.6	1.7	−1.3	8.6	−47.4	3.3
	CB5D4	Maryland	34.3	2.3	84.8	2.4	96.4	0.7	64.4	1.7	44.4	3.6	4.5	2.9
	CB5D4-GFP	Maryland	41.8	3.0	85.8	1.7	95.2	1.6	64.7	3.7	48.1	2.8	−14.3	6.9
	HCedar2	Florida	39.7	5.6	86.5	1.8	97.5	0.1	75.0	0.3	54.4	7.6	20.9	3.0
	HTtP14 [FL-6]	Florida	44.0	3.1	82.3	5.1	95.0	0.9	59.1	4.1	53.9	2.2	−1.7	5.0
	TXsc	Texas	10.7	6.8	72.5	3.8	91.5	0.6	68.1	2.3	7.9	10.5	−22.4	8.3
*P. chesapeaki*	A8-4a	Maryland	−47.8	15.6	55.7	6.8	84.1	2.0	88.0	3.3	−12.0	8.2	−96.0	9.0
*P. olseni*	ALG1	Portugal	14.3	14.7	80.4	1.4	94.9	0.7	51.1	1.3	22.5	1.4	10.5	3.2
*P. mediterraneus*	G2	Spain	−41.9	27.4	78.2	1.3	92.6	0.4	57.0	7.5	36.5	10.9	1.2	21.7
*P. honshuensis*	Mie-3G/H8	Japan	−38.5	15.4	86.2	3.3	95.4	1.5	8.8	14.1	64.6	4.5	38.0	5.9

Data expressed as inhibition mean (%).

We also compared all five *Perkinsus* spp. in culture using the panel of six drug-like and probe-like compounds at a concentration within the range of the determined IC_50_ (2 µM). We found that MMV666021, MMV665807 and MMV666102 were active against all five *Perkinsus* spp. Interestingly, some compounds were not equally effective against all the *Perkinsus* spp. *P. chesapeaki* was not sensitive to MMV665941, MMV396719 and MMV006522 at the concentration tested. *Perkinsus chesapeaki* affects both oysters and clams along the East Coast of the USA [5] and recently it was detected in cockles from Europe [10]. Compared to other *Perkinsus* spp., *P. chesapeaki* A8-4a is characterized by making the culture medium acidic [25], [26], which could affect the potency or the uptake of the compounds tested. Both *P. mediterraneus* G2 and *P. honshuensis* Mie-3G/H8 were not sensitive to MMV665941 at the concentration tested; interestingly, they were also less sensitive to MMV666102, a compound that showed a high degree of inhibition in most *P. marinus* strains and in *P. chesapeaki*. This study highlights an unexpected degree of variability between *Perkinsus* spp. A plausible explanation could be variations in the propagation rates and strategies in the *in vitro* culture affecting the uptake of the compounds. For example, some *Perkinsus* spp. in culture appear to be “locked” at the trophozoite stage while other *Perkinsus* spp. continuously zoosporulate [26], [55]. *P. mediterraneus* cell density only increases two- to sixfold over a 6-week period compared to ten- to thirtyfold in *P. marinus* [47]. With the culture medium indicated above, both *P. mediterraneus* G2 and *P. honshuensis* Mie-3G/H8 are characterized by forming large in clumps in culture; whether this phenotype conditions the uptake of the compounds remains to be demonstrated. To answer these questions would require fine-tuning cultures and dedicated experiments outside the scope of this preliminary large screening.

## Conclusions

By taking full advantage of both the open access Malaria Box and having *Perkinsus* spp. in culture, we have identified numerous compounds that affect the *in vitro* proliferation of the parasite. These primary “hits”, if confirmed, would expand the number of available compounds against *P. marinus* fivefold. To determine whether the drugs tested in this study will be effective in treating or preventing *P. marinus* infections in bivalves, we must first find a delivery method that administers an effective dose to the oyster tissue and toxicity to the bivalve hosts and other organisms in the environment [13]. In this study, we have taken an indirect approach for identifying and characterizing geographic phenotypes on the basis of resistance to selected compounds from the Malaria Box. At this point, we do not have an explanation for this variability. Most of the compounds tested have a very low molecular weight and are most likely taken up non-specifically by the parasite transporters [*P. marinus* genome encodes excess in secondary active transporters (41 out of 66 super families) including Major Facilitator Superfamily, Amino Acid/Auxin Permease, and Drug/Metabolite, unpublished data]. This would require more targeted research outside of the scope of this work. Moreover, these findings should also make the scientific community aware that the conclusions may be limited or can change depending on the particular strain used in the study. What the targets are, and the biological functions affected by these compounds in *Perkinsus* spp., remains an open question. Still, *Perkinsus* can be use as a model to ascertain the mechanism of action of the probe-like compounds.

## Supporting Information

Table S1 **List of Medicines for Malaria Venture Malaria Box compounds developed against** ***Plasmodium falciparum*** **3D7 and used in this study (** http://www.mmv.org/research-development/malaria-box-supporting-information **).**
(XLSX)

Table S2 **Percentage of inhibition of** ***Perkinsus marinus*** **trophozoites growth using drug-like compounds and probe-like compounds (20 µM) at day 4 post-exposure.**
(XLSX)

## References

[1] Mackin JG, Owen HM, Collier A (1950) Preliminary note on the occurrence of a new protistan parasite, *Dermocystidium marinum* n. sp., in *Crassostrea virginica* (Gmelin). Science 111: 328–329. 17791737 10.1126/science.111.2883.328

[2] Ford SE, Chintala MM (2006) Northward expansion of a marine parasite: Testing the role of temperature adaptation. J Exp Mar Bio Ecol 339: 226–235.

[3] Ford SE, Smolowitz R (2007) Infection dynamics of an oyster parasite in its newly expanded range. Mar Biol 151: 119–133.

[4] Smolowitz R (2013) A review of current state of knowledge concerning *Perkinsus marinus* effects on *Crassostrea virginica* (Gmelin) (the eastern oyster). Vet Pathol 50: 404–411. 23462867 10.1177/0300985813480806

[5] Pecher WT, Alavi MR, Schott EJ, Fernández-Robledo JA, Roth L, et al (2008) Assessment of the northern distribution range of selected *Perkinsus* species in eastern oysters (*Crassostrea virginica*) and hard clams (*Mercenaria mercenaria*) with the use of PCR-based detection assays. J Parasitol 94: 410–422. 18564742 10.1645/GE-1282.1

[6] Cáceres-Martínez J, Vasquez-Yeomans R, Padilla-Lardizabal G, del Rio Portilla MA (2008) *Perkinsus marinus* in pleasure oyster *Crassostrea corteziensis* from Nayarit, Pacific coast of Mexico. J Invertebr Pathol 99: 66–73. 18423484 10.1016/j.jip.2008.03.005

[7] Bushek D, Allen SK Jr (1996) Races of *Perkinsus marinus* . J Shellfish Res 15: 103–107.

[8] Reece K, Bushek D, Hudson K, Graves J (2001) Geographic distribution of *Perkinsus marinus* genetic strains along the Atlantic and Gulf coasts of the USA. Mar Biol 139: 1047–1055.

[9] Robledo JAF, Wright AC, Marsh AG, Vasta GR (1999) Nucleotide sequence variability in the nontranscribed spacer of the rRNA locus in the oyster parasite *Perkinsus marinus* . J Parasitol 85: 650–656. 10461944

[10] Carrasco N, Rojas M, Aceituno P, Andree KB, Lacuesta B, et al (2014) *Perkinsus chesapeaki* observed in a new host, the European common edible cockle *Cerastoderma edule*, in the Spanish Mediterranean coast. J Invertebr Pathol 117: 56–60. 24525498 10.1016/j.jip.2014.01.009

[11] Fernández Robledo JA, Vasta GR, Record NR (2014) Protozoan parasites of bivalve molluscs: Literature follows culture. PLoS One 9: e100872. 24955977 10.1371/journal.pone.0100872 PMC4067406

[12] Leite MA, Alfonso R, Cancela ML (2011) Herbicides and Protozoan Parasite Growth Control: Implications for New Drug Development. In: M Larramendy, Soloneski, S, editor editors. Herbicides, Theory and Applications. Rijeka, Croatia: InTech. pp. 567–580.

[13] Shridhar S, Hassan K, Sullivan DJ, Vasta GR, Fernández Robledo JA (2013) Quantitative assessment of the proliferation of the protozoan parasite *Perkinsus marinus* using a bioluminescence assay for ATP content. Int J Parsitol: Drug Drug Resist 3: 85–92. 10.1016/j.ijpddr.2013.03.001 PMC3862420 24533297

[14] Keeling PJ (2010) The endosymbiotic origin, diversification and fate of plastids. Philos Trans R Soc Lond B Biol Sci 365: 729–748. 20124341 10.1098/rstb.2009.0103 PMC2817223

[15] Fichera ME, Roos DS (1997) A plastid organelle as a drug target in apicomplexan parasites. Nature 390: 407–409. 9389481 10.1038/37132

[16] Spangenberg T, Burrows JN, Kowalczyk P, McDonald S, Wells TN, et al (2013) The open access malaria box: a drug discovery catalyst for neglected diseases. PLoS One 8: e62906. 23798988 10.1371/journal.pone.0062906 PMC3684613

[17] Duffy S, Avery VM (2013) Identification of inhibitors of *Plasmodium falciparum* gametocyte development. Malar J 12: 408. 24206914 10.1186/1475-2875-12-408 PMC3842684

[18] Lucantoni L, Duffy S, Adjalley SH, Fidock DA, Avery VM (2013) Identification of MMV malaria box inhibitors of *Plasmodium falciparum* early-stage gametocytes using a luciferase-based high-throughput assay. Antimicrob Agents Chemother 57: 6050–6062. 24060871 10.1128/AAC.00870-13 PMC3837862

[19] Bowman JD, Merino EF, Brooks CF, Striepen B, Carlier PR, et al (2014) Antiapicoplast and gametocytocidal screening to identify the mechanisms of action of compounds within the Malaria Box. Antimicrob Agents Chemother 58: 811–819. 24247137 10.1128/AAC.01500-13 PMC3910863

[20] Bessoff K, Spangenberg T, Foderaro JE, Jumani RS, Ward GE, et al (2014) Identification of *Cryptosporidium parvum* active chemical series by Repurposing the open access malaria box. Antimicrob Agents Chemother 58: 2731–2739. 24566188 10.1128/AAC.02641-13 PMC3993250

[21] Njuguna JT, von Koschitzky I, Gerhardt H, Lammerhofer M, Choucry A, et al (2014) Target evaluation of deoxyhypusine synthase from *Theileria parva* the neglected animal parasite and its relationship to *Plasmodium* . Bioorg Med Chem pii: S0968-0896(14)00347-2. doi:10.1016/j.bmc.2014.05.007. [Epub ahead of print] 10.1016/j.bmc.2014.05.007 24909679

[22] Gauthier JD, Feig B, Vasta GR (1995) Effect of fetal bovine serum glycoproteins on the *in vitro* proliferation of the oyster parasite *Perkinsus marinus*: Development of a fully defined medium. J Eukaryot Microbiol 42: 307–313. 7496389 10.1111/j.1550-7408.1995.tb01585.x

[23] Fernández-Robledo JA, Lin Z, Vasta GR (2008) Transfection of the protozoan parasite *Perkinsus marinus* . Mol Biochem Parasitol 157: 44–53. 17996961 10.1016/j.molbiopara.2007.09.007

[24] Robledo JA, Nunes PA, Cancela ML, Vasta GR (2002) Development of an *in vitro* clonal culture and characterization of the rDNA locus of *Perkinsus atlanticus*, a protistan parasite of the clam *Tapes decussatus* . J Eukariot Microbiol 49: 414–422. 10.1111/j.1550-7408.2002.tb00221.x 12425530

[25] Coss CA, Robledo JA, Ruiz GM, Vasta GR (2001) Description of *Perkinsus andrewsi* n. sp. isolated from the Baltic clam (*Macoma balthica*) by characterization of the ribosomal RNA locus, and development of a species-specific PCR-based diagnostic assay. J Eukariot Microbiol 48: 52–61. 10.1111/j.1550-7408.2001.tb00415.x 11249193

[26] Coss CA, Robledo JA, Vasta GR (2001) Fine structure of clonally propagated *in vitro* life stages of a *Perkinsus* sp. isolated from the Baltic clam *Macoma balthica* . J Eukariot Microbiol 48: 38–51. 10.1111/j.1550-7408.2001.tb00414.x 11249192

[27] Casas SM, Grau A, Reece KS, Apakupakul K, Azevedo C, et al (2004) *Perkinsus mediterraneus* n. sp., a protistan parasite of the European flat oyster *Ostrea edulis* from the Balearic Islands, Mediterranean Sea. Dis Aquat Organ 58: 231–244. 15109147 10.3354/dao058231

[28] Gauthier JD, Vasta GR (1993) Continuous *in vitro* culture of the eastern oyster parasite *Perkinsus marinus* . J Invertebr Pathol 62: 321–323.

[29] La Peyre JF, Faisal M, Burreson EM (1993) *In vitro* propagation of the protozoan *Perkinsus marinus*, a pathogen of the eastern oyster, *Crassostrea virginica* . J Eukariot Microbiol 40: 304–310.

[30] Kleinschuster SJ, Swink SL (1993) A simple method for the *in vitro* culture of *Perkinsus marinus* . Nautilus 107: 76–78.

[31] Chen CZ, Kulakova L, Southall N, Marugan JJ, Galkin A, et al (2011) High-throughput *Giardia lamblia* viability assay using bioluminescent ATP content measurements. Antimicrob Agents Chemother 55: 667–675. 21078930 10.1128/AAC.00618-10 PMC3028786

[32] Goodman CD, Su V, McFadden GI (2007) The effects of anti-bacterials on the malaria parasite *Plasmodium falciparum* . Mol Biochem Parasitol 152: 181–191. 17289168 10.1016/j.molbiopara.2007.01.005

[33] Grimberg BT, Mehlotra RK (2011) Expanding the antimalarial drug drsenal-now, but how? Pharmaceuticals (Basel) 4: 681–712. 21625331 10.3390/ph4050681 PMC3102560

[34] Monzote L, Siddiq A (2011) Drug development to protozoan diseases. Open Med Chem J 5: 1–3. 21629506 10.2174/1874104501105010001 PMC3103878

[35] Stelter K, El-Sayed NM, Seeber F (2007) The expression of a plant-type ferredoxin redox system provides molecular evidence for a plastid in the early dinoflagellate *Perkinsus marinus* . Protist 158: 119–130. 17123864 10.1016/j.protis.2006.09.003

[36] Fernández Robledo JA, Caler E, Matsuzaki M, Keeling PJ, Shanmugam D, et al (2011) The search for the missing link: A relic plastid in *Perkinsus*? Int J Parasitol 41: 1217–1229. 21889509 10.1016/j.ijpara.2011.07.008 PMC6810647

[37] Tasumi S, Vasta GR (2007) A galectin of unique domain organization from hemocytes of the eastern oyster (*Crassostrea virginica*) is a receptor for the protistan parasite *Perkinsus marinus* . J Immunol 179: 3086–3098. 17709523 10.4049/jimmunol.179.5.3086

[38] Feng C, Ghosh A, Amin MN, Giomarelli B, Shridhar S, et al (2013) The Galectin CvGal1 from the Eastern oyster (*Crassostrea virginica*) binds to blood group A oligosaccharides on the hemocyte surface. J Biol Chem 288: 24394–24409. 23824193 10.1074/jbc.M113.476531 PMC3750141

[39] Schott EJ, Pecher WT, Okafor F, Vasta GR (2003) The protistan parasite *Perkinsus marinus* is resistant to selected reactive oxygen species. Exp Parasitol 105: 232–240. 14990317 10.1016/j.exppara.2003.12.012

[40] Hohwy M, Spadola L, Lundquist B, Hawtin P, Dahmen J, et al (2008) Novel prostaglandin D synthase inhibitors generated by fragment-based drug design. J Med Chem 51: 2178–2186. 18341273 10.1021/jm701509k

[41] Na BK, Kang JM, Kim TS, Sohn WM (2007) *Plasmodium vivax*: molecular cloning, expression and characterization of glutathione S-transferase. Exp Parasitol 116: 414–418. 17459379 10.1016/j.exppara.2007.02.005

[42] Rojpibulstit P, Kangsadalampai S, Ratanavalachai T, Denduangboripant J, Chavalitshewinkoon-Petmitr P (2004) Glutathione-S-transferases from chloroquine-resistant and -sensitive strains of *Plasmodium falciparum*: what are their differences? Southeast Asian J Trop Med Public Health 35: 292–299. 15691127

[43] Joseph SJ, Fernández-Robledo JA, Gardner MJ, El-Sayed NM, Kuo CH, et al (2010) The Alveolate *Perkinsus marinus*: biological insights from EST gene discovery. BMC Genomics 11: 228. 20374649 10.1186/1471-2164-11-228 PMC2868825

[44] Garreis KA, La Peyre JF, Faisal M (1996) The effects of *Perkinsus marinus* extracellular products and purified proteases on oyster defence parameters *in vitro* . Fish Shellfish Immunol 6: 581–597.

[45] Brown GD, Reece KS (2003) Isolation and characterization of serine protease gene(s) from *Perkinsus marinus* . Dis Aquat Organ 57: 117–126. 14735929 10.3354/dao057117

[46] Faisal M, Schafhauser DY, Garreis KA, Elsayed E, La Peyre JF (1999) Isolation and characterization of *Perkinsus marinus* proteases using bacitracin-sepharose affinity chromatography. Comp Biochem Physiol, B 123B: 417–426.

[47] Casas SM, Reece KS, Li Y, Moss JA, Villalba A, et al (2008) Continuous culture of *Perkinsus mediterraneus*, a parasite of the European flat oyster *Ostrea edulis*, and characterization of its morphology, propagation, and extracellular proteins *in vitro* . J Eukaryot Microbiol 55: 34–43. 18251801 10.1111/j.1550-7408.2008.00301.x

[48] He Y, Yu H, Bao Z, Zhang Q, Guo X (2012) Mutation in promoter region of a serine protease inhibitor confers *Perkinsus marinus* resistance in the eastern oyster (*Crassostrea virginica*). Fish Shellfish Immunol 33: 411–417. 22683517 10.1016/j.fsi.2012.05.028

[49] Yu H, He Y, Wang X, Zhang Q, Bao Z, et al (2011) Polymorphism in a serine protease inhibitor gene and its association with disease resistance in the eastern oyster (*Crassostrea virginica* Gmelin). Fish Shellfish Immunol 30: 757–762. 21215804 10.1016/j.fsi.2010.12.015

[50] Perkins FO, Menzel RW (1966) Morphological and cultural stages in the life cycle of *Dermocystidium marinum* . Proc Natl Shellfish Assoc 56: 23–30.

[51] Bushek D, Allen SK Jr (1996) Host-parasite interactions among broadly distributed populations of the eastern oyster *Crassostrea virginica* and the protozoan *Perkinsus marinus* . Mar Ecol Prog Ser 139: 127–141.

[52] Panko C, Volety A, Encomio V, Barreto J (2006) Evaluation of the antimalarial drug quinine as a potential chemotherapeutic agent for the eastern oyster parasite, *Perkinsus marinus* . J Shellfish Res 25: 760.

[53] Thompson PC, Rosenthal BM, Hare MP (2011) An evolutionary legacy of sex and clonal reproduction in the protistan oyster parasite *Perkinsus marinus* . Infect Genet Evol 11: 598–609. 21256249 10.1016/j.meegid.2011.01.008

[54] Sibley LD, Mordue DG, Su C, Robben PM, Howe DK (2002) Genetic approaches to studying virulence and pathogenesis in *Toxoplasma gondii* . Philos Trans R Soc Lond B Biol Sci 357: 81–88. 11839185 10.1098/rstb.2001.1017 PMC1692920

[55] Casas SM, La Peyre JF (2013) Identifying factors inducing trophozoite differentiation into hypnospores in *Perkinsus* species. Eur J Protistol 49: 201–209. 22999495 10.1016/j.ejop.2012.07.004

[56] Dungan CF, Reece KS (2006) *In vitro* propagation of two *Perkinsus* spp. parasites from Japanese Manila clams *Venerupis philippinarum* and description of *Perkinsus honshuensis* n. sp. J Eukaryot Microbiol 53: 316–326. 16968449 10.1111/j.1550-7408.2006.00120.x

[57] Blackbourn J, Bower SM, Meyer GR (1998) *Perkinsus qugwadi* sp.nov. (*incertae sedis*), a pathogenic protozoan parasite of Japanese scallops, *Patinopecten yessoensis*, cultured in British Columbia, Canada. Can J Zool Rev Can Zool 76: 942–953.

[58] Moss JA, Xiao J, Dungan CF, Reece KS (2008) Description of *Perkinsus beihaiensis* n. sp., a new *Perkinsus* sp. parasite in oysters of Southern China. J Eukaryot Microbiol 55: 117–130. 18318865 10.1111/j.1550-7408.2008.00314.x

